# Gait Training with Functional Electrical Stimulation Improves Mobility in People Post-Stroke

**DOI:** 10.3390/ijerph20095728

**Published:** 2023-05-05

**Authors:** Maria Tereza Artero Prado Dantas, Deborah Cristina Gonçalves Luiz Fernani, Talita Dias da Silva, Iramaia Salomão Alexandre de Assis, Augusto Cesinando de Carvalho, Sidney Benedito Silva, Luiz Carlos de Abreu, Fabio Augusto Barbieri, Carlos Bandeira de Mello Monteiro

**Affiliations:** 1 Laboratory Design and Scientific Writing, Department of Basic Sciences, ABC Faculty of Medicine, Santo André 09060-650, Brazil; 2 School of Arts, Sciences and Humanities, University of São Paulo (EACH/USP), São Paulo 03828-000, Brazil; 3 Course of Physiotherapy, University of West Paulista (UNOESTE), Presidente Prudente 19050-920, Brazil; 4 Postgraduate Program in Medicine (Cardiology) at Paulista School of Medicine, Federal University of São Paulo (EPM/UNIFESP), São Paulo 04024-002, Brazil; 5 Faculty of Medicine, University City of Sao Paulo (UNICID), São Paulo 03071-000, Brazil; 6 Department of Physical Education, Human Movement Research Laboratory (MOVI-LAB), São Paulo State University (UNESP), Bauru 17033-360, Brazil; 7 Department of Physiotherapy, São Paulo State University (UNESP), Presidente Prudente 19060-900, Brazil; 8 Department of Integrated Health Education, Federal University of Espírito Santo (UFES), Vitória 29040-090, Brazil

**Keywords:** electrical stimulation, gait, mobility, hemiparesis, stroke

## Abstract

(1) Background: Stroke is one of the leading causes of disability. To identify the best treatment strategies for people with stroke (PwS), the aim of the current study was to compare the effects of training on a treadmill with functional electrical stimulation (TT-FES) with training on a treadmill (TT), and to analyze the effects of sequence of training on mobility and the parameters of walking ability. (2) Methods: Prospective, longitudinal, randomized and crossover study, in which 28 PwS were distributed into groups, namely the A-B Group (TT-FES followed by TT) and B-A Group (TT followed by TT-FES), using the foot drop stimulator, and were measured with functional tests. (3) Results: We found improved mobility, balance, non-paretic limb coordination, and endurance only in the group that started with TT-FES. However, sensorimotor function improved regardless of the order of training, and paretic limb coordination only improved in the B-A Group, but after TT-FES. These data indicate that the order of the protocols changed the results. (4) Conclusions: Although biomechanical evaluation methods were not used, which can be considered a limitation, our results showed that TT-FES was superior to isolated training on a treadmill with regard to balance, endurance capacity, and coordination of the non-paretic limb.

## 1. Introduction

Stroke is one of the main causes of death and disability in adults worldwide [1,2], leading to motor [3], cognitive [4], and sensorial impairments [5], including hemiparesis, which is a hallmark of the disease. People with stroke (PwS) may present dorsiflexor weakness and foot drop in the paretic lower limb [6], decreasing their ability to walk and leading to higher incidences of stumbling and falling [7,8]. In this sense, gait rehabilitation is a good strategy to improve mobility in PwS, and treadmill training is one of the main methods that can be adopted [9,10,11,12], among other forms of training [13,14,15,16]. Mobility involves some parameters of lower limb coordination, balance, endurance, sensorimotor function, walking ability, and other capacities [17,18], and training these parameters in PwS may promote mobility benefits. For example, it is reported in the literature that the improved mobility after treadmill training in PwS is due to the increasing gait speed, better balance, and higher endurance [19,20].

However, repetitive motor activity alone, such as treadmill training, may not be sufficient to produce representational plasticity in cortical motor maps [21]. As a result, the use of orthoses and other equipment during the execution of training are strategies to increase the benefits of treadmill training in PwS. Nevertheless, a large number of patients are unable to use orthoses during training [22], due to complaints about comfort, weight, difficulty of use, and the appearance of the orthosis [23], and thus one possibility that could minimize these limitations of the orthosis, as well as providing gait assistance, by producing neural changes and increasing the positive effects of treadmill training, is the use of functional electrical stimulation (FES).

FES is a promising therapy to facilitate neural mechanisms and provide motor recovery [24]. In theory, as the device promotes electrically induced contractions, which, combined with voluntary contractions, are able to strengthen spinal synapses and provoke cortical alterations [25], the combination of FES and gait rehabilitation appears to represent an efficient treatment for PwS [26]. When current is applied to the fibular region, the dorsiflexor paretic musculature is electrically activated while the initial swing and posture phase of the gait cycle occurs, resulting in “active” foot elevation. In fact, the use of this electrical current promotes modifications in conduction velocity, axonal growth, and myelination of peripheral nerves [27]. Furthermore, the central effects of FES allow for peripheral efferent activation, with greater functionality in the strength of the contractions and resistance to muscle fatigue [28], as well as increased muscle mass [29] and coordination of movement [30]. Therefore, FES is able to modify the control movement through the promotion of motor relearning, by providing a pathway for synchronized presynaptic and postsynaptic activity [31].

In this sense, a recent possibility for providing FES for lower limbs in PwS is the use of a foot drop stimulator. This equipment can be used throughout the day when walking in the community to promote greater quality of movement during gait. The device provides a tilt sensor and accelerometer that induce ankle dorsiflexion during gait, improving range of motion, muscle strength, and gait pattern [31]. A foot drop stimulator was used in this study as this equipment allows for the automatic triggering of the electrical stimulus at the exact moment of the beginning of dorsiflexion during the gait cycle. Therefore, the use of FES as a foot drop stimulator leads to movements closer to physiological gait, and when there are changes in this cycle pattern, adjustments are made to the electrical stimuli of the equipment itself, facilitating gait training [32,33,34], even on the treadmill.

Considering the above, we organized a protocol to compare the effects of treadmill training with FES (TT-FES) and treadmill training in isolation (TT). Therefore, we verified the influence of sequence of training (i.e., one group started using TT-FES and changed to TT, and the other group practices the opposite sequence) on mobility and important parameters of walking ability (e.g., sensorimotor function, balance, limb coordination, and endurance) in PwS.

We hypothesized that both kinds of training would improve mobility and walking ability, but that TT-FES would have a large effect compared with TT. In addition, training first with the sequence of training with the TT-FES would positively influence the subsequent practice (with treadmill training only).

## 2. Materials and Methods

### 2.1. Participants

The individuals in the sample were recruited from rehabilitation centers in Presidente Prudente (São Paulo, Brazil). The inclusion criteria were a medical diagnosis of post-stroke sequelae, with motor conditions that use treadmill training associated with electrical stimulation, and a time of more than three months since the lesion. The exclusion criteria were the presence of surgery or chemical neuromuscular blockade in the lower limbs in the six months prior to participation in the study, and osteoarticular deformities that prevent independent walking.

In total, 51 PwS who did not use any type of orthoses participated in this study, of which 28 individuals completed the protocol. All participants showed foot drop during the swing phase of the gait cycle, due to stroke sequelae, but with preserved passive range of motion of the ankle joint, which was active only without the action of gravity. However, these elements of stroke sequelae were not part of the outcomes directly analyzed in this research, which included the functional effects of the protocol applied to gait with this characteristic.

Furthermore, there was sample loss was due to 12 individuals who presented cardiac problems (high cardiac frequency, hypertension, uncontrolled arrhythmias, and coronary obstruction); meanwhile, there were three individuals who presented cognitive deficits and were not able to perform the sensorimotor assessments; one individual left the training; and seven demonstrated severe motor deficits that made physical training impossible. However, the 28 individuals who completed the protocol were representative of the population of post-stroke patients in the rehabilitation centers of the Western Paulista region who had the same characteristics. Not all results can be attributed to the global population with this condition.

The participants were distributed into two groups according to the type of training (TT-FES or TT), and following a quasi-experimental design, each participant recruited was allocated to one of the groups according to sex, age, and compromised body side, in order to maintain the homogeneity of the groups. The individual characterization data are presented in Table 1 and the comparisons between groups are presented in Table 2.

### 2.2. Design and Instruments

**Figure 1:**
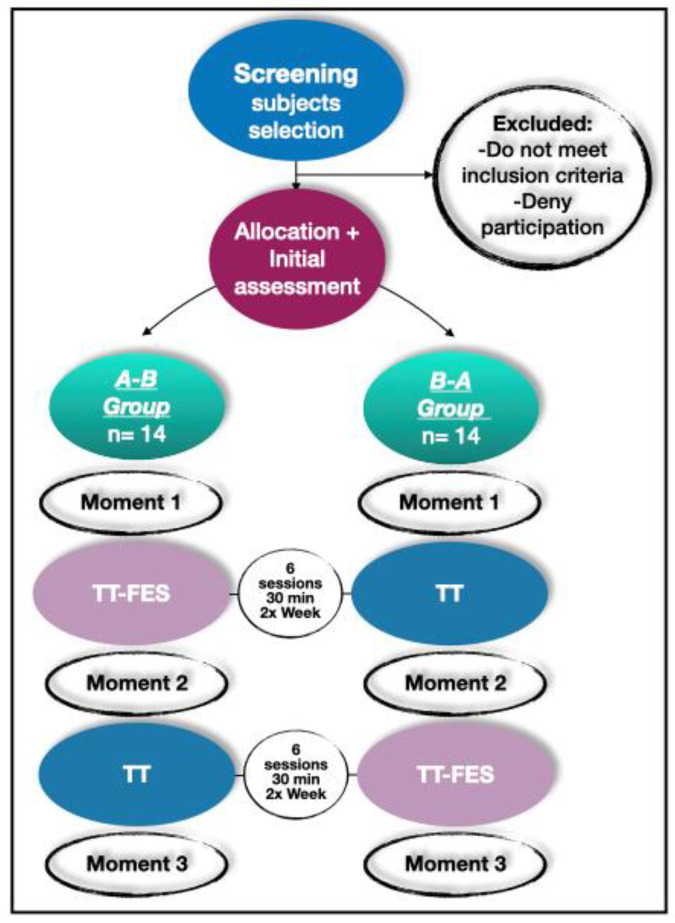
Flowchart of group sequence of training. TT-FES: treadmill sequence of training with the use of FES; TT: treadmill sequence of training without electrical stimulation.

In the current study, to examine the effects of treadmill training with FES, we proposed a longitudinal, randomized, crossover trial, including two treadmill sequences of training, which differed in the order of FES use between the groups. The sequence of training was composed of six sessions, twice a week, with a frequency of 30 min, and a total of 12 sessions. For this, PwS were distributed into two groups: Group A-B started the sequence of training with treadmill with the use of FES (TT-FES) and in the middle of the training they changed to treadmill without stimulation (TT), while Group B-A started with only TT and in the middle of the training they changed to TT-FES. Functional tests were performed to evaluate both groups before starting the sequence of training (Moment 1), after six sessions of the first sequence (Moment 2), and after six sessions of the second sequence of training (Moment 3) (Figure 1).

The time required to perform the 10 Meter Walk Test (10MWT) was used to determine the initial velocity/intensity of the treadmill for the training (40% of result was selected for programming the velocity of training until reaching the training heart rate). Moreover, to ensure safety for participants during training, we designated a maximum limit (submaximal heart rate) for each participant.

For the protocol with FES, the commercially available WalkAide^®^ (Innovative Neurotronics, Austin, TX, USA), was used as the foot-drop stimulator. The stimulator has a tilt sensor and accelerometer, which induces ankle dorsiflexion and facilitates control of the duration of nerve stimulation during the swing phase of gait. We decided to use WalkAide^®^ due to the efficacy of the instrument observed in the studies of Everaert et al. [32], Bethoux et al. [33], and Bethoux et al. [34]. These studies included the same device (WalkAide^®^) and population (PwS); however, during deambulation in activities of daily living (comparing WA use with conventional ankle–foot orthosis (AFO)) and not in functional training (treadmill walking with FES). Moreover, the authors emphasized that both devices (WA and AFO) produced similar functional improvements, but that the execution of studies that approach FES in tasks that involve functional mobility is important [32,33,34], as in the training protocol executed in our study.

Before and after performing the sessions, participants remained seated for analysis of the cardiorespiratory parameters (blood pressure, oxygen saturation, heart and respiratory rates). The heart rate and Modified Borg Scale were monitored constantly during training.

### 2.3. Outcome Measures

The individuals were evaluated at three moments (Figure 1). Mobility (main outcome of this study) was evaluated by the Timed Up and Go Test (TUG) and 10MWT. In addition, sensory-motor impairment (Fugl-Meyer Assessment—FMA), balance (Berg Balance Scale—BBS), lower limb coordination (Lower Extremity Motor Coordination Test—LEMOCOT), and endurance functional capacity (6-Minute Walk Test—6MWT) were evaluated. Moreover, to evaluate any influence of cognition in the protocol, we used the Mini-Mental State Examination (MMSE) to assess cognitive function.

### 2.4. Data Analysis

To identify homogeneity between groups, a paired *t*-test and chi-square test were used with body mass, height, BMI, age, time of lesion, MMSE, sex, type of stroke, and affected side of the body. Moreover, to compare moments 1, 2, and 3, another paired *t*-test was carried out using scores from the 10MWT, TUG, and 6MWT between TT-FES and TT.

The dependent variables used were the scores in the BBS, FMA, LEMOCOT (paretic lower limb), and LEMOCOT (non-paretic lower limb), the time to complete the 10MWT and TUG, and the distance in 6MWT. The dependent variables used were submitted to a MANOVA with factor 2 (Groups A-B and B-A) by 3 (moments 1, 2, and 3), with repeated measures on the factors. Partial eta-squared (η^2^) was reported to measure effect size and interpreted as small (effect size > 0.01), medium (effect size > 0.06), or large (effect size > 0.14). Post hoc comparisons were carried out using the Tukey’s HSD test. The software package used was SPSS, 20.0 (significance was maintained at 0.05).

## 3. Results

There were no differences between groups for the anthropometric data, age, time of lesion, MMSE, sex, type of stroke, and affected side of body, demonstrating the homogeneity of the sample (Table 2).

The MANOVA revealed a significant effect for moments, F(7, 14) = 4.18, *p* < 0.001; Wilks’ lambda = 0.288. The separate RM-ANOVAs for each test are reported in the following sections. Even though the MANOVA did not find significant effects or interactions for the groups, we reported the effects for groups found in the separate ANOVAs. Table 3 presents a summary of the significant results of the analysis of variance, and the means and standard deviations for each parameter are presented in Table 4.

### 3.1. Moment 1 vs. Moment 2

Both groups presented improved sensorimotor impairments (FMA) after the first six sessions. However, only the TT-FES was able to largely improve mobility (TUG), balance (BBS), endurance capacity (6MWT), and coordination of the non-paretic limb (LEMOCOT) after the first part of the sequence of training.

### 3.2. Moment 2 vs. Moment 3

No significant effects were found.

### 3.3. Moment 1 vs. Moment 3

Both groups presented improved performance for sensorimotor function (FMA), balance (BBS), coordination of the non-paretic limb (LEMOCOT), and endurance capacity (6MWT). However, only the B-A group (started with TT) presented improved coordination of the paretic limb (LEMOCOT) and only the A-B group (started with TT-FES) presented improved mobility (TUG) (Table 3 and Table 4).

## 4. Discussion

The current study aimed to verify the effects of an FES protocol on treadmill training in PwS. Our hypotheses were partially verified, participants presented improved mobility and parameters of walking ability (balance, non-paretic limb coordination, and endurance) only in the group that started with TT-FES. However, sensorimotor function improved independent of the order of training, and paretic limb coordination only improved in the group that started with TT, but after training with TT-FES. These data indicate that the order of the protocols changed the results. Therefore, in the following paragraphs, we will discuss possible explanations for the positive effects of treadmill training with and without FES application, considering the dependent variables.

### 4.1. Sensorimotor Function

The treadmill training (independently of FES application) showed an improvement in PwS (see Table 3 FMA data). On the other hand, the order of the protocol did not change the results, as the use of FES and treadmill training combined did not promote an improvement in sensorimotor function when compared with the treadmill training without FES. In this context, another study used the same instrument (FMA) to assess individuals with post-stroke who underwent training on a treadmill, and also found an improvement in motor function, which emphasizes the benefits of gait training on a treadmill [35].

According to our findings, treadmill training can be considered an efficient intervention in PwS for sensorimotor function. A possible explanation for this is that gait exercises can prevent contractures in lower limb joints, increase endurance and muscle strength, and delay or prevent the appearance of spasticity, so that these gains lead to an increase in walking function [36]. In addition, treadmill training exposes the central nervous system to multiple sources of conflicting sensory information, constantly challenging sensory reweighting processes, as well as proprioceptive inputs from the lower extremities. This practice provides an appropriate stride pattern on a mobile support surface with benefits to gait function [37].

### 4.2. Balance

Positive results were found in BBS when comparing before and after the intervention in PwS. However, the order of the protocols changed the results, as these benefits were observed only when FES was used associated with training on a treadmill, as the B-A Group only demonstrated improvement in balance after the second protocol, performed with FES (see Table 3 bulletin board data), while the A-B Group demonstrated an improvement after the first protocol.

A systematic review showed the importance and benefits of treadmill training for balance in PwS, which could be due to the reverse transfer associated with the possibility of performing walking training at higher intensities and dosages than in many applied interventions [12]. However, the results found emphasize that the use of FES for balance during treadmill walking in PwS provides a positive improvement, but only from the combined intervention (treadmill and FES), and it should be considered as an interesting option for rehabilitation.

Supporting the outcomes of this research, Robertson et al. [38] analyzed balance with BBS after stroke, using walking on the ground training with and without FES (using the same device used in the current study), and found an improvement in balance in the FES group. Despite the benefits, however, the authors pointed out that the use of FES while walking on the ground could cause deterioration in the individual’s confidence in relation to their balance, until they adapt to the use of the equipment. However, the improvement in the lower limb range of motion during the swing phase with the use of FES over time promotes greater confidence in the individual and probably improves balance. Thus, it is possible to speculate that, in our intervention group, the use of gait training with FES provided sensory integration stimuli and this challenging situation may also have translated into better scores in BBS [37].

### 4.3. Endurance Capacity

In addition to balance, with regard to endurance capacity, the association of FES with training on a treadmill also showed superior results compared with the exclusive use of the treadmill, indicating that the order of the protocols changed the results. For endurance, the A-B Group demonstrated improvement after the first and second protocols, while the B-A Group only obtained satisfactory results after the second protocol, when FES was used. This indicates not only the effectiveness of FES therapy combined with treadmill training, but also its superiority over the second option alone.

In this same way, it is important to mention another study that also used WalkAide^®^ and assessed walking resistance through 6MWT [34]. The authors point out that although participants with stroke treated with FES did not demonstrate a sufficient magnitude of change to meet the established criteria for significant change (50 m) [39], the improvement in these individuals represented a clinically relevant change [34]; a 13% change in the 6MWT was adopted as indicative of true clinical change [40]. In view of this, in another study, an increase in distance of 16.3% (28.6 m) was observed [39].

With respect to these considerations, the results of Bethoux et al. [34] assume that, even though the highest range of significant change was not reached, the 6MWT findings demonstrate real improvements in the subjects’ endurance capacity. In view of this, the outcomes of the study cited [34] and those of the current research converge on the understanding that FES promotes satisfactory effects on the endurance capacity of individuals with stroke and should be used combined with training on a treadmill, so that the subject achieves better responses from the treatment.

### 4.4. Motor Coordination

LEMOCOT was used to verify the influence of treadmill training on coordination and the impact on both lower limbs, even with the application of FES only on the paretic lower limb. The results for coordination of the paretic lower limb showed improvement only in the B-A Group, after the completion of the second protocol, when FES was used, demonstrating that the order of the protocols changed the results.

However, considering the coordination of the non-paretic lower limb, the data showed improvement after both the protocols, but only when FES was included with the treadmill training. In other research, the authors reported that interventions that involve both lower limbs, such as treadmill training, improve at least the non-paretic lower limb and, consequently, develop the gait function [41]. Thus, it is possible to speculate that the different stimuli provided by the use of FES in the paretic lower limb provided an adaptation to the walking task. In addition, to maintain gait function, the non-paretic lower limb needed to adjust in order to adapt to this new demand, which may have been responsible for the improvement in lower limb coordination. Furthermore, disuse, with reduced levels of physical activity in PwS combined with increased task demand may also have influenced the outcome related to the coordination of both limbs [41].

This difference in outcomes between groups may be due to the need for an initial period of training on the treadmill (without FES), for an individual to adapt to the gait task. In this case, the B-A Group had this moment of adaptation before using FES, and took advantage of the resource in a more satisfactory way, while the A-B Group did not receive this “advantage”. Thus, the improvement in lower limb motor coordination in the B-A Group occurred after the application of FES, but with a period of previous practical experience. The use of FES for motor relearning in stroke patients requires intensive and prolonged therapy, and this needs to be adapted to the individual to allow for increased use of the paretic limb [42].

In this sense, the use of FES for the coordination of the paretic lower limb is still very controversial. Kautz et al. [43] described that, for most individuals post-stroke, there was no progress in post-treatment FES coordination. However, the use of functional stimulation with intramuscular electrodes showed an improvement in coordination [44]. In this context, the improvement in impaired motor coordination is not clear when it is associated with FES during an activity, such as leg cycle ergometry or treadmill training [36]. Although benefits have been found with the use of a treadmill and FES intervention to improve coordination movements, there may be a need for practical adaptation to the task before starting stimulation. Therefore, the use of FES and treadmill to improve lower limb motor coordination, should be further studied in the future.

### 4.5. Mobility

To evaluate the improvement in walking functionality in PwS, we used different locomotor tests, namely 10MWT to assess walking speed and TUG to assess mobility.

However, no significant effects of the TT-FES (or TT) were found during 10MWT, which can be considered an index of functionality, disability, and quality of life in PwS [33]. The individuals presented similar values (between 0.6 and 1.1 m/s), that is, the individuals who trained without FES presented initial 10MWT values of 1.06 ± 0.30 m/s and final values of 1.17 ± 0.42 m/s, and the group that executed the training with FES presented initial values of 0.62 ± 0.41 m/s and final values of 0.72 ± 0.46 m/s at the three moments analyzed. Moreover, another study that used FES with individuals post-stroke found a significant improvement in gait velocity [33]; however, the initial mean in the 10MWT of individuals in those studies was generally lower than the value presented in the current study.

Contrary to our findings, Sheffler et al. [45] emphasized that the use of FES or usual care (ankle–foot orthosis or no device) during gait training in rehabilitation demonstrated significant improvements in walking speed, capacity, functional mobility, and quality of life. Furthermore, previous studies using FES with PwS found a significant improvement in gait speed [33]. The explanation for the behavior of these data is that the post-stroke participants already had a reasonable community walking score (see Table 4, 10MWT data), according to the classification whereby 0.4 to 0.8 m/s indicates limited community walking, and above 0.8 m/s indicates unlimited community walking [46]. A good walking ability before starting the protocol indicates the need for a longer training period than the one used in this research in order to improve gait speed.

The effects of the intervention in the A-B Group were beneficial in the results of mobility (TUG), as this group presented an improvement after the first and second protocols, while the B-A Group showed no improvement. Thus, the order of the protocols led to a difference in the mobility outcome. The reduction in spasticity, improvement in ankle dorsiflexion, and the increase in neural plasticity may explain the positive impacts of the FES on mobility [47]. Robertson et al. [38] suggested that training including walking on different surfaces with FES increased confidence compared with training without FES, improving balance (similar to our findings) and, consequently, walking ability.

### 4.6. Limitation and Future Studies

Two limitations should be presented: (1) a short intervention period, all participants performed 12 sessions, of which only 6 included FES, and future studies should include a protocol with a longer period of intervention, and (2) we did not analyze other quantitative and qualitative elements, such as step count and gait kinematic analysis, which could provide better information for discussion.

## 5. Conclusions

Important results for the rehabilitation of PwS were found, especially with regard to treadmill training and the effects of FES on mobility and important parameters for locomotion. Treadmill training was sufficient to improve the sensorimotor function of PwS. However, the effect of FES combined with treadmill training was superior to isolated training on a treadmill with regard to balance, endurance capacity, and coordination of the non-paretic limb, which are important variables for mobility in individuals with stroke.

## Figures and Tables

**Table 1:** Individual characterization data according to groups.

	Type of Stroke	Time of Lesion (Months)	Affected Side of Body	Body Mass (kg)	Height (m)	BMI (kg/m^2^)	Age (Years)	Sex	MMSE (Points)
A-B Group	I	44	R	75.3	1.59	29.7	61	F	23
	I	14	R	58.2	1.54	24.5	60	F	26
	H	3	R	65.1	1.62	24.8	45	F	20
	I	25	L	73.0	1.62	27.8	42	F	28
	H	38	L	43.0	1.50	19.1	44	F	28
	I	31	R	65.5	1.70	22.6	70	M	20
	I	5	R	70.0	1.80	21.6	58	M	26
	I	120	L	78.2	1.68	27.7	57	M	19
	I	24	L	73.0	1.68	25.8	71	M	18
	I	3	L	66.0	1.62	25.1	53	M	22
	I	38	L	78.0	1.63	29.3	77	M	25
	I	168	L	91.0	1.69	31.8	69	M	28
	H	38	L	91.0	1.73	30.4	47	M	28
	H	15	L	90.0	1.78	28.4	34	M	6
B-A Group	H	299	R	63.5	1.47	29.3	65	F	18
	H	83	R	60.0	1.75	19.5	51	F	17
	H	185	R	59.0	1.55	23.5	62	F	18
	I	5	L	87.2	1.57	35.3	64	F	13
	I	61	L	64.0	1.58	25.6	52	F	29
	H	185	L	72.0	1.75	23.5	59	F	29
	I	58	R	89.5	1.78	28.2	66	M	26
	I	60	R	73.0	1.65	26.8	69	M	25
	I	126	R	106.3	1.80	32.8	39	M	20
	H	36	R	67.0	1.81	20.4	24	M	18
	I	29	L	64.0	1.65	23.5	75	M	26
	I	16	L	70.0	1.74	23.1	71	M	30
	I	23	L	75.0	1.66	27.2	61	M	25
	I	26	L	99.0	1.65	36.3	51	M	24

A-B Group, initial protocol treadmill training with stimulation followed by treadmill training without stimulation; B-A Group, initial protocol treadmill training without stimulation followed by treadmill training with stimulation; H, hemorrhagic; I, ischemic; R, right; L, left; F, female; M, male.

**Table 2:** Sample characterization.

	A-B Group		B-A Group		
	Mean	SD	Mean	SD	*p*-Values *
Body Mass (kg)	72.7	12.8	75.0	14.3	0.669
Height (m)	1.7	0.1	1.7	0.1	0.650
BMI (kg/m^2^)	26.2	4.9	26.3	3.6	0.752
Age (years)	56.3	12.3	57.8	13.1	0.766
Time of lesion (months)	40.4	45.3	85.1	81.7	0.096
MMSE (points)	21.3	8.5	22.7	5.1	0.619
	n		n		*p*-Values **
Sex	5 women 9 men		6 women 8 men		1
Type of stroke	10 ischemic 4 hemorrhagic		9 ischemic 5 hemorrhagic		1
Affected side of body	5 right 9 left		7 right 7 left		0.704

A-B Group, initial protocol treadmill training with stimulation followed by treadmill training without stimulation; B-A Group, initial protocol treadmill training without stimulation followed by treadmill training with stimulation; BMI, body mass index; MMSE, mini mental state examination; SD, standard deviation; n, sample size. * *t*-test; ** chi-square test.

**Table 3:** A summary of significant results of the analysis of variance, considering the analysis within moments in each group and between groups (TT-FES and TT). The arrows indicate that the TT-FES presented better results considering all moments together (effect for Groups) compared with TT in the LEMOCOT (paretic limb), TUG, and 6MWT. The opposite occurred for TUG, with no differences between groups for FMA, BBS, and LEMOCOT (non-paretic limb).

	Comparison within Moments							Comparison between Groups		
	A-B (Started with TT-FES)			B-A (Started with TT)			Effect for Moments	A-B	B-A	Effects for Groups
	1 vs. 2	2 vs. 3	1 vs. 3	1 vs. 2	2 vs. 3	1 vs. 3		Started with TT-FES	Started with TT	
FMA	*p* = 0.008	-	*p* = 0.017	*p* = 0.001	-	*p* = 0.035	$F(2,\;40)=7.41,\;p=0.002,\;\eta_{p}^{2}$= 0.27	-	-	-
BBS	*p* = 0.012	-	*p* = 0.003	-	-	*p* = 0.035	$F(2,\;40)=8.64,\;p=0.003,\;\eta_{p}^{2}$= 0.30	-	-	-
LEMOCOT (non-paretic limb)	*p* = 0.005	-	*p* = 0.001	-	-	*p* = 0.015	$F(2,\;40)=12.6,\;p\;<\;0.001,\;\eta_{p}^{2}$= 0.39	-	-	-
LEMOCOT (paretic limb)	-	-	-	-	-	*p* = 0.026	$F(2,\;40)=3.98,\;p=0.028,\;\eta_{p}^{2}$= 0.06	↓	↑	$F(1,\;20)=5.89,\;p=0.025,\;\eta_{p}^{2}$= 0.23
10MWT	-	-	-	-	-	-	-	↑	↓	$F(1,\;20)=3.67,\;p=0.070,\;\eta_{p}^{2}$= 0.16
TUG	*p* = 0.004	-	*p* < 0.001	-	-	-	$F(2,\;40)=10.9,\;p=0.001,\;\eta_{p}^{2}$= 0.35	↓	↑	$F(1,\;20)=7.16,\;p=0.015,\;\eta_{p}^{2}$= 0.26
6MWT	*p* = 0.029	-	*p* = 0.032	-	-	*p* = 0.005	F(2, 40) = 9.52, *p* = 0.001, $\eta_{p}^{2}$= 0.32	↓	↑	$F(1,\;20)=4.00,\;p=0.059,\;\eta_{p}^{2}$= 0.17

A-B Group, initial protocol treadmill training with stimulation followed by treadmill training without stimulation; B-A Group, initial protocol treadmill training without stimulation followed by treadmill training with stimulation; vs., versus; FMA, Fugl-Meyer Assessment; BBS, Berg Balance Scale; LEMOCOT, Lower Motor Coordination Test; 10 MWT, 10-Meter Walk Test; TUG, Time Up and Go Test; 6MWT, 6-Minute Walk Test; FES, Functional Electrical Stimulation; Moments, 1: baseline assessment, 2: assessment after 6 interventions; 3 assessment after 12 interventions and crossover; TT-FES, treadmill sequence of training with the use of FES; TT treadmill sequence of training without electrical stimulation.

**Table 4:** Means and standard deviations for parameters evaluated for both groups at the three assessment moments and the predicted values of the LEMOCOT and 6MWT tests.

	Moment 1		Moment 2		Moment 3		Predicted Value		Percentage Improvement Moment 1 vs. 2		Percentage Improvement Moment 1 vs. 3	
	A-B Mean (SE) [95% CI]	B-A Mean (SE) [95% CI]	A-B Mean (SE) [95% CI]	B-A Mean (SE) [95% CI]	A-B Mean (SE) [95% CI]	B-A Mean (SE) [95% CI]	A-B Mean (SE)	B-A Mean (SE)	A-B	B-A	A-B	B-A
FMA (%)	73.1 (5.9) [60.7–85.4]	77.5 (5.4) [66.2–88.7]	77.6 (5.2) [66.8–88.4]	83.1 (4.7) [73.3–92.9]	80.4 (4.8) [70.3–90.5]	83.3(4.4) [74.1–92.5]	-	-	5.8%	6.7%	9.1%	7.0%
BBS (points)	48.9 (0.9) [47.1–50.7]	51.5 (0.8) [49.8–53.2]	51.8 (1.1) [49.5–54.2]	52.8 (1.0) [50.6–54.9]	52.1 (0.7) [50.6–53.6]	53.4 (0.7) [52.0–54.8]	-	-	5.6%	2.5%	6.1%	3.5%
LEMOCOT (non-paretic limb) (hits)	19.6 (2.6) [14.1–25.1]	25.1 (2.4) [20.1–30.2]	27.8 (3.1) [21.3–34.3]	29.9 (2.8) [23.9–35.9]	30.4 (3.1) [23.9–36.8]	31.5 (2.8) [25.6–37.4]	33.9 (1.3)	33.7 (1.6)	29.5%	16.1%	35.5%	20.3%
LEMOCOT (paretic limb) (hits)	7.5 (2.3) [2.7–12.3]	14.9 (2.1) [10.5–19.3]	10.3 (3.0) [4.1–16.5]	18.8 (2.7) [13.1–24.4]	10.3 (2.9) [4.2–16.4]	19.0 (2.7) [13.4–24.6]	32.2 (1.2)	32.0 (1.5)	27.2%	20.7%	27.2%	21.6%
10MWT (m/s)	0.7 (0.1) [0.5–1.0]	1.0 (0.1) [0.8–1.2]	0.8 (0.1) [0.6–1.1]	1.1 (0.1) [0.9–1.4]	0.9 (0.1) [0.6–1.2]	1.1 (0.1) [0.8–1.4]	-	-	12.5%	9.1%	22.2%	9.1%
TUG (s)	21.8 (2.9) [15.7–27.8]	11.7 (2.6) [6.2–17.3]	16.9 (1.7) [13.5–20.3]	10.5 (1.5) [7.4–13.7]	16.5 (1.9) [12.6–20.4]	10.1 (1.7) [6.6–13.7]	-	-	22.5%	10.3%	24.3%	13.7%
6MWT (m)	183.7 (32.9) [115.0–252.4]	272.6 (30.1) [209.9–335.3]	230.5 (35.2) [157.2–303.9]	305.3 (32.1) [238.3–372.2]	239.3 (37.3) [161.4–317.2]	343.1 (34.1) [272.0–414.2]	544.7 (13.2)	544.5 (15.7)	20.3%	10.7%	23.3%	20.5%

SE, Standard error; CI, Confidence interval; vs., versus; A-B Group, initial protocol treadmill training with stimulation followed by treadmill training without stimulation; B-A Group, initial protocol treadmill training without stimulation followed by treadmill training with stimulation; BBS, Berg Balance Scale; FMA, Fugl-Meyer Assessment; LEMOCOT, Lower Motor Coordination Test; 10MWT, 10-Meter Walk Test; TUG, Time Up and Go Test; 6MWT, 6-Minute Walk Test.

## Data Availability

The data presented in this study are available upon request from the corresponding author. The data are not publicly available due to other studies in progress.
